# Supplementary material for: Spatial metabolomics reveals glycogen as an actionable target for pulmonary fibrosis
Source: Nat Commun. 2023 May 13;14:2759. doi: 10.1038/s41467-023-38437-1 (PMC10182559; doi:10.1038/s41467-023-38437-1)
Supplement: Supplementary file 1 — Supplementary information [file 41467_2023_38437_MOESM1_ESM.pdf]

Supplementary information

Spatial metabolomics reveals glycogen as an actionable target for pulmonary fibrosis

Lindsey R. Conroy[1,2,#], Harrison A. Clarke[3,#], Derek B. Allison[2,4,#], Samuel Santos Valenca[5,6,#], Qi Sun[1], Tara R. Hawkinson[3], Lyndsay E.A. Young[7], Jelena A. Juras[1,2], Juanita E. Ferreira[4], Autumn V. Hammonds[4], Jaclyn B. Dunne[8], Robert J. McDonald[4], Kimberly J. Absher[4], Brittany E. Dong[5,6], Ronald C. Bruntz[7], Kia H. Markussen[7], Warren J. Alilain[1,9], Jinze Liu[10], Matthew S. Gentry[2,3,7,11], Peggi M. Angel[8], Christopher M. Waters[5,6,*], and Ramon C. Sun[1,2,3,11,*]

Affiliations:

[1]Department of Neuroscience, University of Kentucky College of Medicine, Lexington, KY 40536-0298, USA
[2]Markey Cancer Center, Lexington, KY 40536-0298, USA
[3]Department of Biochemistry & Molecular Biology, College of Medicine, University of Florida, Gainesville, FL, USA
[4]Department of Pathology and Laboratory Medicine, University of Kentucky College of Medicine, Lexington, KY 40536, USA
[5]Department of Physiology, University of Kentucky College of Medicine, Lexington, KY 40536, USA
[6]Saha Cardiovascular Research Center, University of Kentucky, Lexington, KY 40536, USA
[7]Department of Molecular and Cellular Biochemistry, University of Kentucky College of Medicine, Lexington, KY 40536-0298, USA
[8]Department of Cell & Molecular Pharmacology & Experimental Therapeutics at the Medical University of South Carolina
[9]Spinal Cord and Brain Injury Research Center
[10]Department of Biostatistics, Massey Cancer Center, Virginia commonwealth university, Richmond, VA 23284, USA
[11]Center for Advanced Spatial Biomolecule Research, University of Florida, Gainesville, FL, USA
# These authors contributed equally: Lindsey R. Conroy; Harrison A. Clarke; Derek B. Allison; Samuel Santos Valenca
* These authors jointly supervised this work: Christopher M. Waters; Ramon C. Sun
Correspondence: chris.waters41@uky.edu; ramonsun@ufl.edu

Running title: Glycogen is required for pulmonary fibrosis progression.

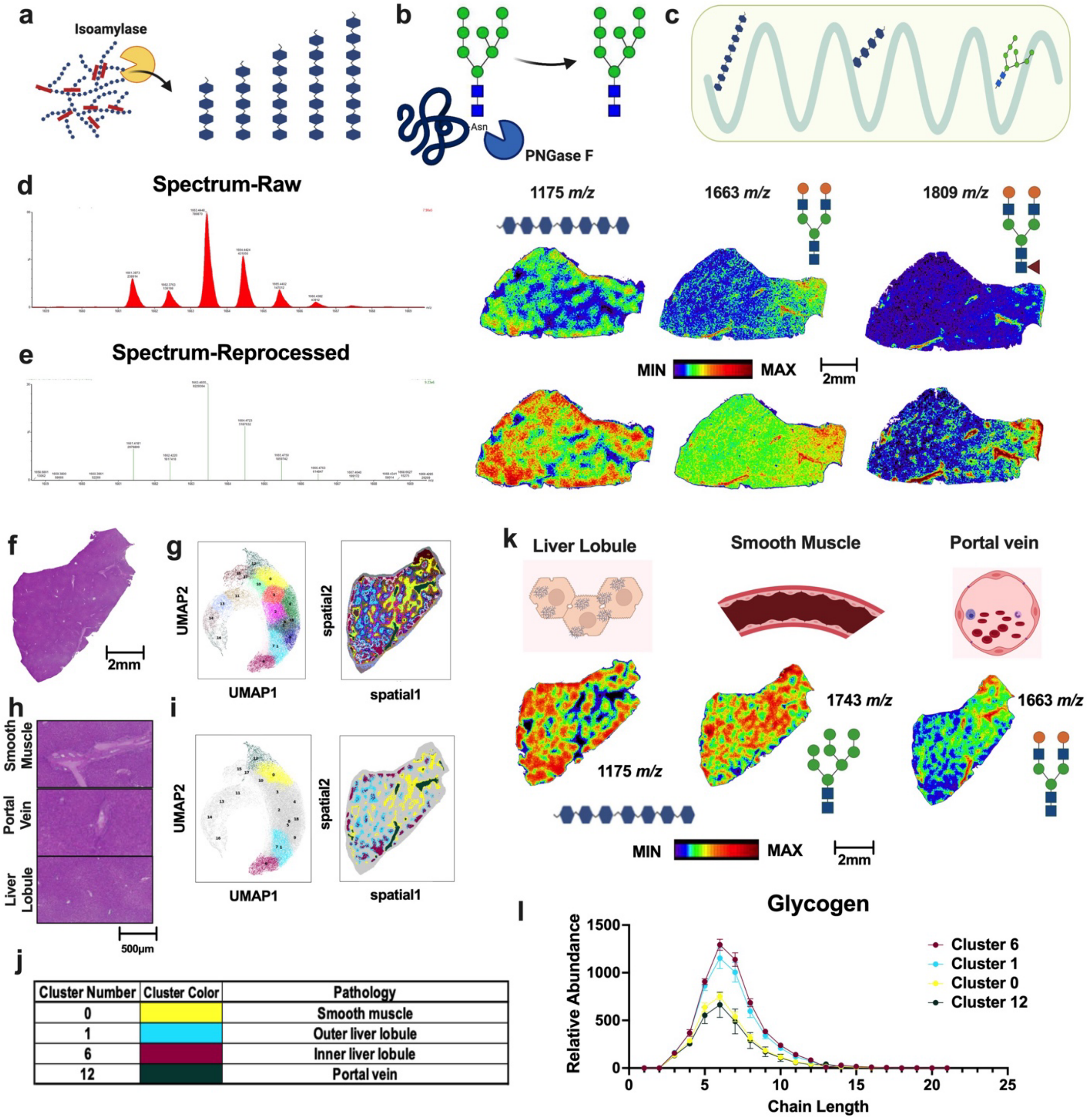
a
Isoamylase
b
Asn
PNGase F
c
d
Spectrum-Raw
e
Spectrum-Reprocessed
1175 m/z
1663 m/z
1809 m/z
MIN
MAX
2mm
f
2mm
g
UMAP2
UMAP1
spatial2
spatial1
h
Smooth Muscle
Portal Vein
Liver Lobule
500μm
i
UMAP2
UMAP1
spatial2
spatial1
j
Cluster Number | Cluster Color | Pathology
0 | | Smooth muscle
1 | | Outer liver lobule
6 | | Inner liver lobule
12 | | Portal vein
k
Liver Lobule
Smooth Muscle
Portal vein
1175 m/z
1743 m/z
1663 m/z
MIN
MAX
2mm
l
Glycogen
Relative Abundance
Chain Length
Cluster 6
Cluster 1
Cluster 0
Cluster 12
0
500
1000
1500
0
5
10
15
20
25

Supplementary Figure 1.

High-dimensionality reduction and spatial clustering (HDR-SC) analysis reveals liver tissue architecture and metabolism.

a Schematic of isoamylase digestion of glycogen to produce free linear oligosaccharide chains. Created with BioRender.com. **b** Schematic of peptide: N-glycosidase F (PNGase F) release of free N-linked glycans from glycoproteins. Created with BioRender.com. **c** Schematic of ion mobility separation of linear oligosaccharide chains and N-linked glycans during MALDI-MSI. Created with BioRender.com. **d** Representative unprocessed and combined ion spectra showing mass drift during MALDI-MSI (left) and spatial heatmap of representative linear oligosaccharide and N-glycans using unprocessed *m/z*. Scale bar: 2mm. **e** Representative combined ion spectra post processing accounting for mass drift during MALDI-MSI (left) and spatial heatmap of representative linear oligosaccharide and N-glycans using post processed *m/z*. Scale bar: 2mm. **f** Hematoxylin and eosin (H&E) staining of an immediate adjacent section used for MALDI-MSI for histopathology assessment. **g** All identified clusters visualized by UMAP (left) and spatial plots (right) based on pixel coordinate information. **h** Zoomed in images of H&E staining of liver sections showing liver lobules, smooth muscle, and portal veins. **i** UMAP (left) and spatial (right) plots highlighting inner liver lobule (cluster 6), outer liver lobule (cluster 1), smooth muscle (cluster 0), and portal vein (cluster 12). **j** Annotation of spatial clusters to histopathology by a panel of board-certified pathologists. **k** Schematics of liver architecture regions (top) and representative spatial distribution and heatmap of a single carbohydrate feature for each region based on differential expression analysis. Molecular structure and corresponding *m/z* (rounded to the nearest whole number) of each complex carbohydrate feature were shown below the spatial heatmap. Scale bar: 2mm. **l** Glycogen chain length abundance and distribution based on UMAP clusters between inner liver lobule (cluster 6), outer liver lobule (cluster 1), smooth muscle (cluster 0), and portal vein (cluster 12). Values are presented as mean +/- standard error. n=3 technical replicates per cluster.

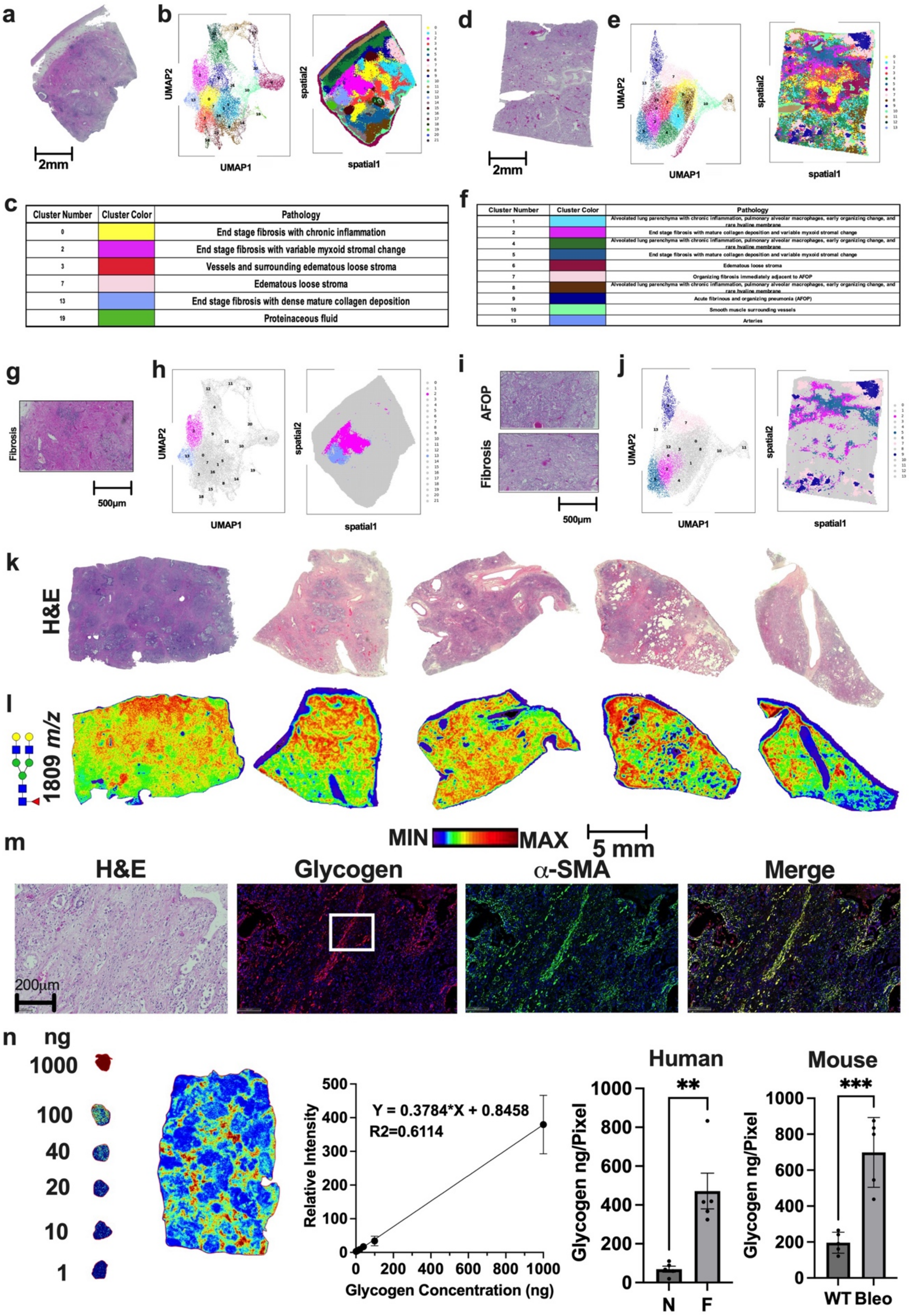
a
2mm
b
UMAP2
UMAP1
spatial2
spatial1
c
Cluster Number | Cluster Color | Pathology
0 | End stage fibrosis with chronic inflammation
2 | End stage fibrosis with variable myxoid stromal change
3 | Vessels and surrounding edematous loose stroma
7 | Edematous loose stroma
13 | End stage fibrosis with dense mature collagen deposition
19 | Proteinaceous fluid
d
2mm
e
UMAP2
UMAP1
spatial2
spatial1
f
Cluster Number | Cluster Color | Pathology
1 | Alveolated lung parenchyma with chronic inflammation, pulmonary alveolar macrophages, early organizing change, and rare hyaline membrane
2 | End stage fibrosis with mature collagen deposition and variable myxoid stromal change
4 | Alveolated lung parenchyma with chronic inflammation, pulmonary alveolar macrophages, early organizing change, and rare hyaline membrane
5 | End stage fibrosis with mature collagen deposition and variable myxoid stromal change
6 | Edematous loose stroma
7 | Organizing fibrosis immediately adjacent to AFOP
8 | Alveolated lung parenchyma with chronic inflammation, pulmonary alveolar macrophages, early organizing change, and rare hyaline membrane
9 | Acute fibrinous and organizing pneumonia (AFOP)
10 | Smooth muscle surrounding vessels
13 | Arteries
g
Fibrosis
500µm
h
UMAP2
UMAP1
spatial2
spatial1
i
AFOP
Fibrosis
500µm
j
UMAP2
UMAP1
spatial2
spatial1
k
H&E
l
1809 m/z
MIN
MAX
5 mm
m
H&E
Glycogen
α-SMA
Merge
200µm
n
ng
1000
100
40
20
10
1
Y = 0.3784*X + 0.8458
R2=0.6114
Relative Intensity
Glycogen Concentration (ng)
Human
**
Glycogen ng/Pixel
N
F
Mouse
***
Glycogen ng/Pixel
WT
Bleo

Supplementary Figure 2.
High-dimensionality reduction and spatial clustering (HDR-SC) analysis of tissue sections from idiopathic pulmonary fibrosis (IPF) and COVID-19 patients.
a Hematoxylin and eosin (H&E) staining of an immediate adjacent IPF section used for MALDI-MSI for histopathology assessment. **b** All identified clusters visualized by UMAP (left) and spatial plots (right) based on pixel coordinate information. **c** Annotation of spatial clusters to histopathology by a panel of board-certified pathologists. **d** H&E staining of an immediate adjacent COVID-19 section used for MALDI-MSI for histopathology assessment. **e** All identified clusters visualized by UMAP (left) and spatial plots (right) based on pixel coordinate information. **f** Annotation of spatial clusters to histopathology by a panel of board-certified pathologists. **g** Zoomed in images of H&E staining of IPF section showing end-stage fibrosis. Scale bar: 500μm. **h** UMAP (left) and spatial (right) plots highlighting end-stage fibrosis clusters. **i** Zoomed in image of H&E staining of COVID-19 section showing acute fibrinous organizing pneumonia (AFOP) and end-stage fibrosis. Scale bar: 500μm. **j** UMAP (left) and spatial (right) plots highlighting AFOP and end-stage fibrosis clusters. **k** H&E staining of additional IPF (n=3, left) and COVID-19 (n=2) tissue sections used for MALDI-MSI for histopathology assessment. Scale bar is below in **l**. **l** Spatial distribution and heatmap of N-linked glycan 1809 *m/z* in additional patient tissues shown in **k**. **m** Immunofluorescent/co-localization analysis of glycogen and alpha smooth muscle actin (α-SMA) from an adjacent 20μm section of IPF specimen shown in **Fig. 2a**. Tissue is stained with glycogen (red), α-SMA (green), and DAPI (blue) following by whole slide scanning and visualized using the HALO software. Zoomed in field of view (Square) is shown in **Fig. 2o**. **n** Quantitative glycogen MALDI imaging in situ. Increasing amounts of purified glycogen were spotted adjacent to the tissue section and used to generate the standard curve. Absolute glycogen levels in fibrosis and non-fibrotic regions were determined using the equation derived from the line of best fit (Human: n=5 normal and fibrosis patients; Mouse: n=5 animals/group). Values are presented as mean +/- standard error. *p*-value were calculated using two-tailed *t*-test.

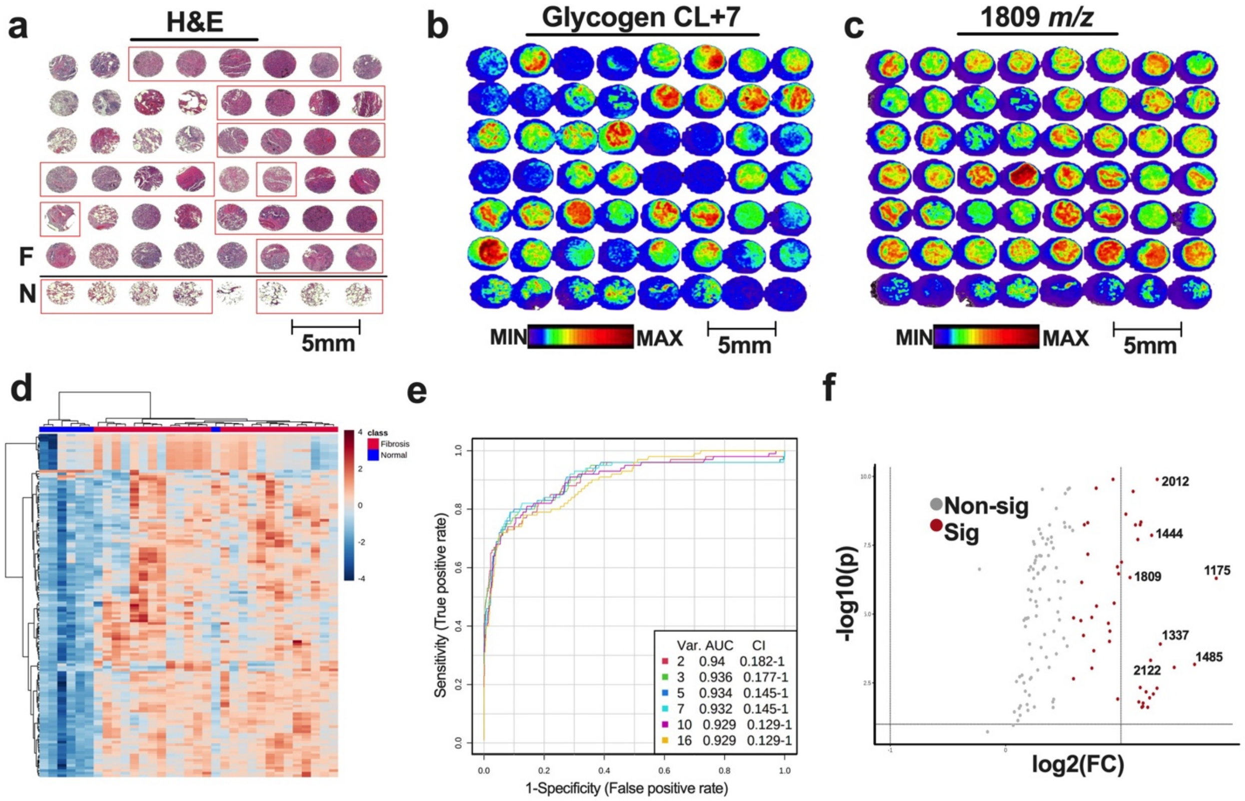

Supplementary Figure 3.

Pulmonary fibrosis patient tissue exhibits increased glycogen accumulation and unique N-linked glycosylation profiles compared to normal lung.

a Hematoxylin & Eosin (H&E) staining of the pulmonary fibrosis TMA used for this study with normal (N) and fibrotic (F) tissues annotated. Tissue cores used were further confirmed by pathologist and used in the analysis are highlighted in red. Scale bar: 5mm. **b** Spatial distribution and heatmap of glycogen chain length +7 (1175 *m/z*) in pulmonary fibrosis TMA shown in **a**. Scale bar: 5mm. **c** Spatial distribution and heatmap of N-linked glycan 1809 *m/z* in pulmonary fibrosis TMA shown in **a**. Scale bar: 5mm. **d** Unsupervised clustering heatmap analysis of all glycogen and N-linked glycan features in normal and fibrosis lung patient samples. **e** Multivariate receiver operating characteristic (ROC) curve of all glycogen features between normal and fibrosis patients. **f** Volcano plot of the fold change between fibrosis/normal lung patient tissue. Glycogen and N-linked glycans features with a fold change>1.5 and $P<0.1$ are highlighted in red. *p*-value were calculated using multiple two-tailed *t*-test followed by Benjamini, Krieger, and Yekutieli FDR.

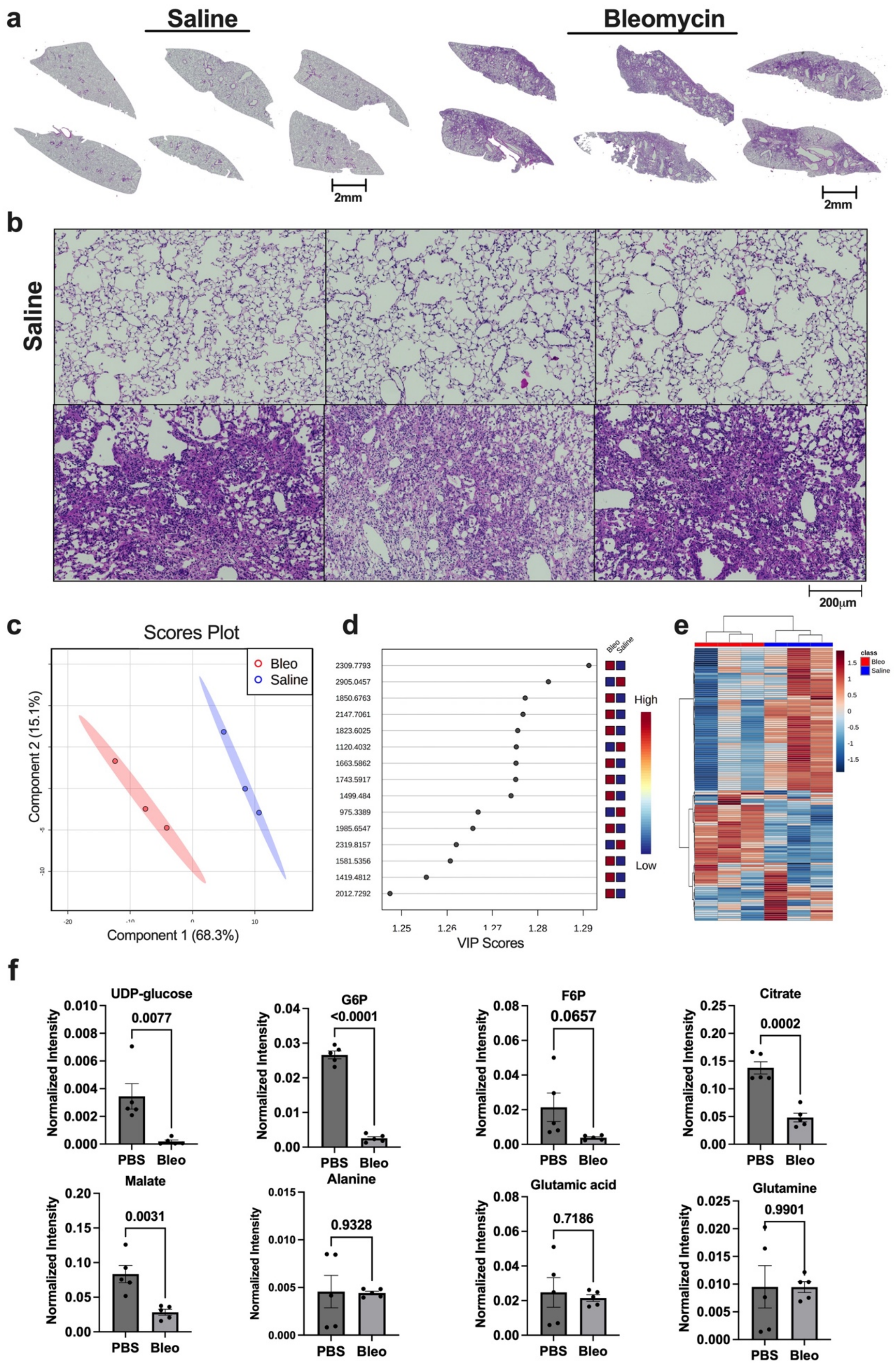
a
Saline
Bleomycin
2mm
2mm
b
Saline
200μm
c
Scores Plot
Bleo
Saline
Component 2 (15.1%)
Component 1 (68.3%)
d
Bleo
Saline
2309.7793
2905.0457
1850.6763
2147.7061
1823.6025
1120.4032
1663.5862
1743.5917
1499.484
975.3389
1985.6547
2319.8157
1581.5356
1419.4812
2012.7292
High
Low
1.25
1.26
1.27
1.28
1.29
VIP Scores
e
class
Bleo
Saline
1.5
1
0.5
0
-0.5
-1
-1.5
f
UDP-glucose
0.0077
G6P
<0.0001
F6P
0.0657
Citrate
0.0002
Malate
0.0031
Alanine
0.9328
Glutamic acid
0.7186
Glutamine
0.9901
Normalized Intensity
PBS
Bleo

Supplementary Figure 4.
Bleomycin-treatment induces aberrant complex carbohydrate metabolism during fibrosis *in vivo*.
a Hematoxylin and eosin (H&E) staining of whole lung tissue from control (saline, left) and bleomycin-treated (right) mice. Scale bar: 2mm. **b** Zoomed in representative images of H&E staining of lung tissue from control (saline, top) and bleomycin-treated (bottom) mice. Scale bar: 200µm. **c** Multivariate analysis of glycogen and N-linked glycan features in lung tissue from control (saline) and bleomycin-treated mice by partial least squares-discriminant analysis (PLS-DA) displaying 95% confidence intervals. **d** Variable importance in projection (VIP) analysis showing top 15 most discriminant carbohydrate structures revealed by PLS-DA. *m/z* can be matched to structure in Supplemental Table 1. **e** Unsupervised clustering heatmap analysis of all glycogen and N-linked glycan features in lung tissue from control (saline) and bleomycin-treated mice. **f** Targeted liquid-chromatography mass spectrometry analysis of polar metabolites from PBS and bleomycin treated mice (n=5 animals/group). Values are presented as mean +/- standard error. *p*-value were calculated using two-tailed *t*-test.

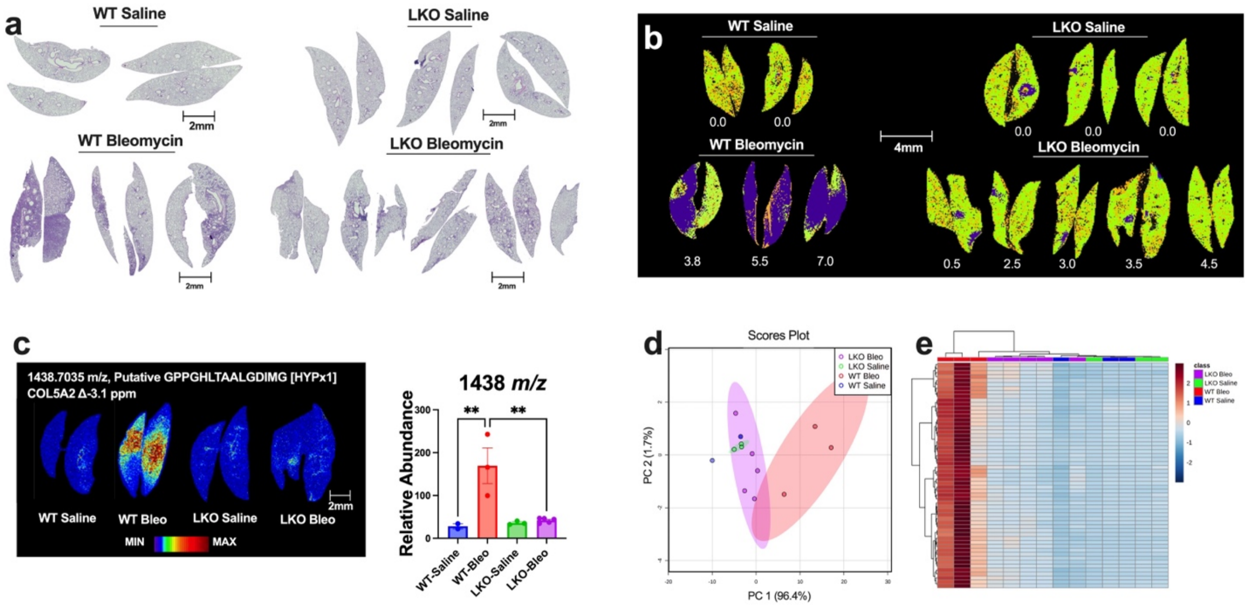

Supplementary Figure 5.
Loss of Laforin blunts bleomycin-induced aberrant complex carbohydrate metabolism *in vivo*.
a Hematoxylin and eosin (H&E) staining of whole lung tissue from control (saline) and bleomycin-treated wild-type (WT) and *Epm2a*$^{-/-}$ (LKO) mice. Scale bar: 2mm. **b** Total collagen peptides detected from WT, LKO, WT-Bleomycin, and Laforin-KO-Bleomycin lung by MALDI-MSI. Ashcroft score is listed below for each set of mouse lungs. Scale bar: 2mm. **c** Spatial distribution and heatmap of collagen peptide 1438 *m/z* in lung tissue from control (saline) and bleomycin-treated WT and LKO mice and total abundance of 1438 *m/z* in lung tissue from control (saline) and bleomycin-treated WT and LKO mice (n=2 animals for WT-saline, n=3-5 animals for other groups). Putative amino acid sequence, collagen subtype, and # of hydroxylate proline sites (HYP) on are displayed on top. Scale bar: 2mm. Values are presented as mean +/- standard error. *p*-value were calculated using one-way ANOVA followed by multiple comparisons test. **d** Multivariate analysis of collagen peptides in lung tissue from control (saline) and bleomycin-treated WT and Laforin-KO mice by principal component analysis (PCA) displaying 95% confidence intervals. **e** Unsupervised clustering heatmap analysis of all collagen peptides in lung tissue from control (saline) and bleomycin-treated WT and LKO mice.

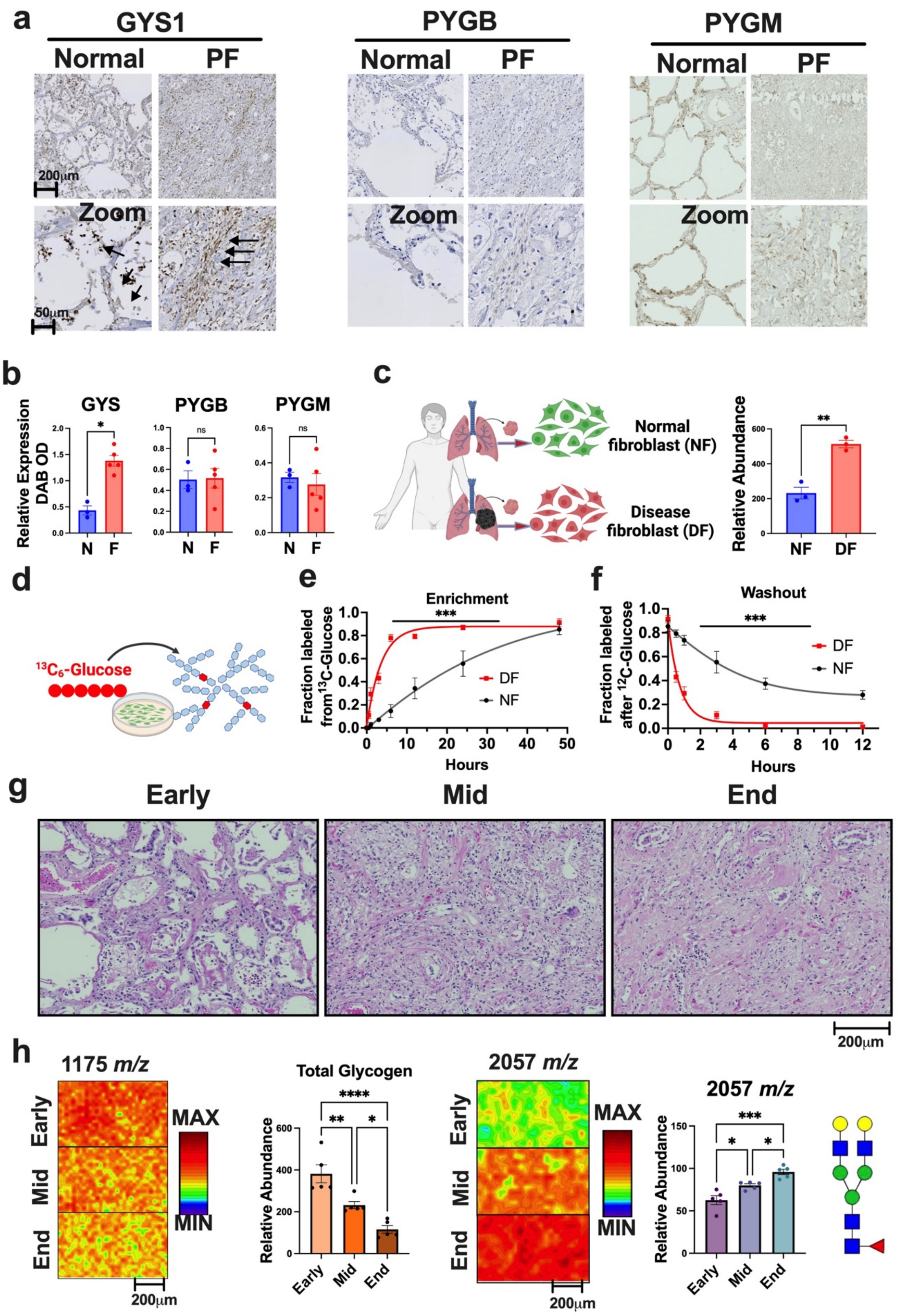
a
GYS1
Normal
PF
200μm
Zoom
50μm
PYGB
Normal
PF
Zoom
PYGM
Normal
PF
Zoom
b
Relative Expression DAB OD
GYS
PYGB
PYGM
N
F
c
Normal fibroblast (NF)
Disease fibroblast (DF)
Relative Abundance
NF
DF
d
¹³C₆-Glucose
e
Enrichment
Fraction labeled from ¹³C-Glucose
DF
NF
Hours
f
Washout
Fraction labeled after ¹²C-Glucose
DF
NF
Hours
g
Early
Mid
End
200μm
h
1175 m/z
Early
Mid
End
MAX
MIN
200μm
Total Glycogen
Relative Abundance
2057 m/z
MAX
MIN
200μm
2057 m/z
Relative Abundance

Supplementary Figure 6.
Increased metabolic demand for glycogen in diseased fibroblasts.
a Representative immunohistochemical staining of glycogen synthase (GYS1), glycogen phosphorylate brain isoform (PYGB), glycogen phosphorylate muscle isoform (PYGM) in fibrotic regions and adjacent alveoli structures. Zoomed in images are at the bottom. Scale bar: 200µm and 50µm. Arrows represent alveoli lining and fibrosis with GYS1 staining. **b** Quantification of immunohistochemical staining analysis for GYS, PYGB, PYGM abundance in fibrotic regions (n=5 human patient samples) and adjacent normal regions where available (n =3 human patient samples). Values are presented as mean +/- standard error. *p*-value were calculated using two-tailed *t*-test. **c** Left: schematics of patient-derived normal fibroblasts (NF) and disease fibroblasts (DF, isolated from IPF patients). Right: Relative glycogen levels between NF and DF measured by GCMS (n=3 repeat experiments). Values are presented as mean +/- standard error. *p*-value were calculated using two-tailed *t*-test. Created with BioRender.com. **d** Schematics of ^{13}C-glucose tracing to study glycogen biosynthesis and degradation. Created with BioRender.com. **e** ^{13}C-glycogen enrichment from ^{13}C-glucose over 48 hours in both NF and DF. (n=3 independent repeats/group for each time point). Values are presented as mean +/- standard error *p*-value were calculated using two-way ANOVA following by multiple comparisons testing. **f** ^{13}C-glycogen washout after ^{12}C-glucose substitution over 12 hours in both NF and DF. (n=3 independent repeats/group for each time point). Values are presented as mean +/- standard error *p*-value were calculated using two-way ANOVA following by multiple comparisons testing. **g** Hematoxylin and eosin (H&E) staining showing early, mid, and end stage fibrosis in human PF specimens. **h** Representative images and relative abundance of glycogen chain length +7 (1175 *m/z*) and a biantennary glycan spatial distribution in in early-, mid-, and end-stage fibrosis. Molecular structure of the selected N-linked glycan is to the right of the heatmap (n= 5 unique ROI within each group; each ROI is >100 pixel). Values are presented as mean +/- standard error. *p*-value were calculated using one-way ANOVA followed by multiple comparisons test.

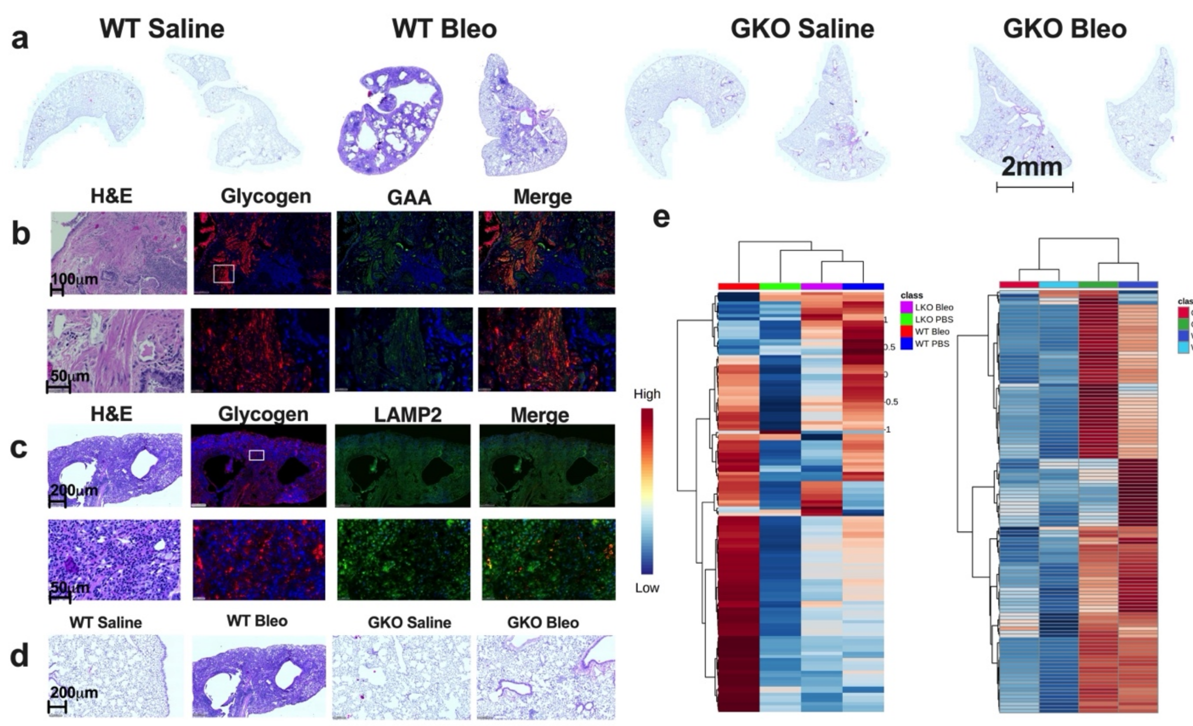

Supplementary Figure 7.

Gaa$^{-/-}$ is protective against bleomycin-induced lung injury *in vivo*.

a Hematoxylin and eosin (H&E) staining of whole lung tissue from control (saline) and bleomycin-treated wild-type (WT) and Gaa$^{-/-}$ (GKO) mice. Scale bar: 2mm. **b** Immunofluorescent/co-localization analysis of glycogen and acid alpha-glucosidase (GAA) from an adjacent 20µm section of human IPF lung shown in Fig. 2a. Tissue is stained with glycogen (red), GAA (green), and DAPI (blue) following by whole slide scanning and visualized using the HALO software. Scale bar: 100µm and 50µm respectively. Zoomed in field of view of the same slide is shown in Fig. 6A. **c** Immunofluorescent/co-localization analysis of glycogen and LAMP2 from an adjacent 20µm section of human IPF lung shown in Fig. 2a. Tissue is stained with glycogen (red), LAMP2 (green), and DAPI (blue) following by whole slide scanning and visualized using the HALO software. Scale bars: 200µm and 50µm respectively. Zoomed in field of view of the same slide is shown in Fig. 6B. **d** Representative images of H&E staining of lung tissue from control (saline) and bleomycin-treated WT and GKO mice. Scale bar: 200µm **e** Unsupervised clustering heatmap analysis of all glycogen and N-linked glycan features in lung tissue from control (saline) and bleomycin-treated WT and LKO/GKO mice.

a

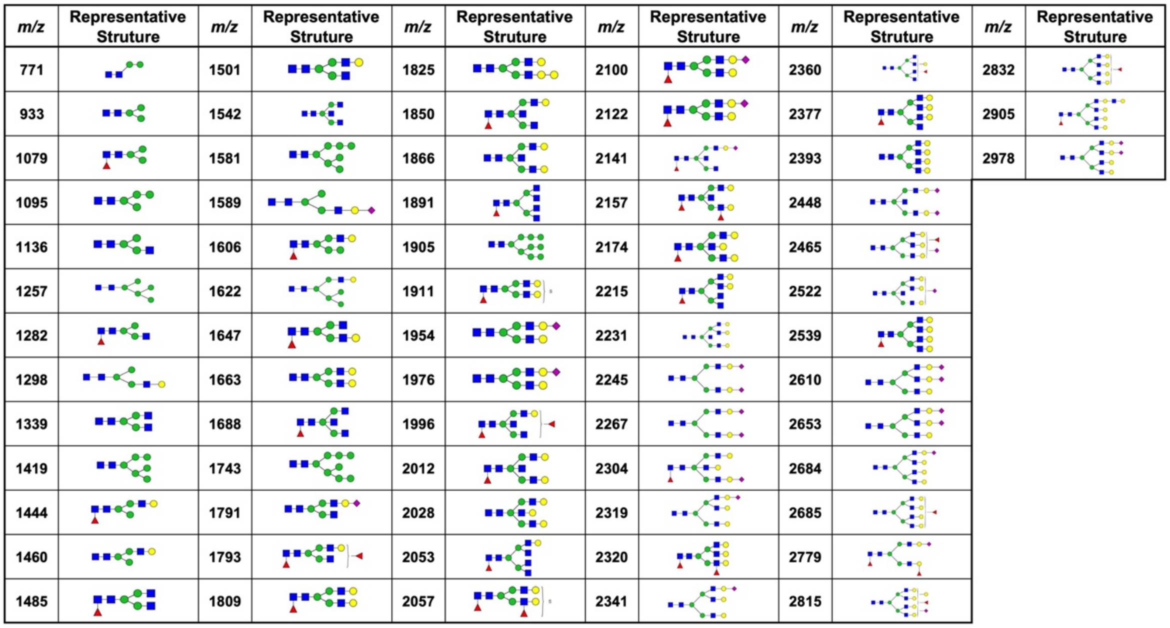

m/z	Representative Struture	m/z	Representative Struture	m/z	Representative Struture	m/z	Representative Struture	m/z	Representative Struture	m/z	Representative Struture
771		1501		1825		2100		2360		2832	
933		1542		1850		2122		2377		2905	
1079		1581		1866		2141		2393		2978	
1095		1589		1891		2157		2448			
1136		1606		1905		2174		2465			
1257		1622		1911		2215		2522			
1282		1647		1954		2231		2539			
1298		1663		1976		2245		2610			
1339		1688		1996		2267		2653			
1419		1743		2012		2304		2684			
1444		1791		2028		2319		2685			
1460		1793		2053		2320		2779			
1485		1809		2057		2341		2815			

b

m/z	527	689	851	1013	1175	1337	1499	1662	1824
Composition	3x	4x	5x	6x	7x	8x	9x	10x	11x
m/z	1986	2148	2310	2472	2634	2797	3121	3443	
Composition	12x	13x	14x	15x	16x	17x	19x	21x	
m/z	569	731	893	1056	1218	1379	1542	1704	1866
Composition	3x + P	4x + P	5x + P	6x + P	7x + P	8x + P	9x + P	10x + P	11x + P
m/z	2028	2189	2351	2514	2676	2838	3000	3162	3324
Composition	12x + P	13x + P	14x + P	15x + P	16x + P	17x + P	18x + P	19x + P	20x + P

Supplementary Table 1.
Matrix-Assisted Laser Desorption/Ionization Mass Spectrometry Imaging (MALDI-MSI) of complex carbohydrates. a Molecular structure of N-linked glycans and their corresponding monoisotopic mass (*m/z*) rounded to the nearest whole number. **b** Molecular structure of representative linear oligosaccharide chains and phospho- linear oligosaccharide chains and their corresponding monoisotopic mass (*m/z*) rounded to the nearest whole number.

Molecule	Formula	Adduct	m/z	Retention Time (min)
UDP-glucose	C15H24N2O17P2	[M-H]-	565.0477	5.612
Malate	C4H6O5	[M-H]-	133.0142	5.546
Glutamic Acid	C5H9NO4	[M-H]-	146.0459	5.745
Alanine	C3H7N1O2	[M-H]-	88.0404	5.387
Glutamine	C5H10N2O3	[M-H]-	145.0619	5.513
Citrate	C6H8O7	[M-H]-	191.0197	4.601
Glucose 6-phosphate	C6H13O9P	[M-H]-	259.0224	6.1
Fructose 6-phosphate	C6H13O9P	[M-H]-	259.0224	6.45

Supplementary Table 2. Annotated metabolites with their molecular formula, adduct, m/z, and retention time.
