# Supplementary material for: Spatial metabolomics reveals glycogen as an actionable target for pulmonary fibrosis
Source: Nat Commun. 2023 May 13;14:2759. doi: 10.1038/s41467-023-38437-1 (PMC10182559; doi:10.1038/s41467-023-38437-1)
Supplement: Supplementary file 3 — Reporting Summary [file 41467_2023_38437_MOESM3_ESM.pdf]

nature portfolio

Corresponding author(s): Ramon Sun

Last updated by author(s): Aug 5, 2022

Reporting Summary

Nature Portfolio wishes to improve the reproducibility of the work that we publish. This form provides structure for consistency and transparency in reporting. For further information on Nature Portfolio policies, see our Editorial Policies and the Editorial Policy Checklist.

Statistics

For all statistical analyses, confirm that the following items are present in the figure legend, table legend, main text, or Methods section.

n/a	Confirmed	
☐	☒	The exact sample size (*n*) for each experimental group/condition, given as a discrete number and unit of measurement
☐	☒	A statement on whether measurements were taken from distinct samples or whether the same sample was measured repeatedly
☐	☒	The statistical test(s) used AND whether they are one- or two-sided<br>*Only common tests should be described solely by name; describe more complex techniques in the Methods section.*
☒	☐	A description of all covariates tested
☒	☐	A description of any assumptions or corrections, such as tests of normality and adjustment for multiple comparisons
☐	☒	A full description of the statistical parameters including central tendency (e.g. means) or other basic estimates (e.g. regression coefficient) AND variation (e.g. standard deviation) or associated estimates of uncertainty (e.g. confidence intervals)
☐	☒	For null hypothesis testing, the test statistic (e.g. *F*, *t*, *r*) with confidence intervals, effect sizes, degrees of freedom and *P* value noted<br>*Give P values as exact values whenever suitable.*
☒	☐	For Bayesian analysis, information on the choice of priors and Markov chain Monte Carlo settings
☒	☐	For hierarchical and complex designs, identification of the appropriate level for tests and full reporting of outcomes
☒	☐	Estimates of effect sizes (e.g. Cohen's *d*, Pearson's *r*), indicating how they were calculated

Our web collection on statistics for biologists contains articles on many of the points above.

Software and code

Policy information about availability of computer code

Data collection	Data was acquired using Waters MassLynx v4.2. Bruker timsControl V4.1.12, Fleximaging V7.2
Data analysis	Data was analyzed using Waters HDI v1.5, GraphPad Prism 9.0, Metaboanalyst 5.0, Halo V3.4.2986, and Python 3.10, SciLS lab V2023a

For manuscripts utilizing custom algorithms or software that are central to the research but not yet described in published literature, software must be made available to editors and reviewers. We strongly encourage code deposition in a community repository (e.g. GitHub). See the Nature Portfolio guidelines for submitting code & software for further information.

Data

Policy information about availability of data

All manuscripts must include a data availability statement. This statement should provide the following information, where applicable:

- Accession codes, unique identifiers, or web links for publicly available datasets
- A description of any restrictions on data availability
- For clinical datasets or third party data, please ensure that the statement adheres to our policy

All MALDI imaging data files; metabolomics data files; are deposited online and free to download without restriction. identifiers are included win the data availability section of the mauscript

Human research participants

Policy information about [studies involving human research participants and Sex and Gender in Research.](#)

Reporting on sex and gender	Sex and gender were not reported for the study.
Population characteristics	Population characteristics were not obtained for the study.
Recruitment	Human research participants were not recruited for the study.
Ethics oversight	De-identified human patient tissues were obtained from the University of Kentucky Biospecimen Procurement and Translation Pathology Shared Resource Facility with Institutional Review Board (IRB) approved exempt status.

Note that full information on the approval of the study protocol must also be provided in the manuscript.

Field-specific reporting

Please select the one below that is the best fit for your research. If you are not sure, read the appropriate sections before making your selection.

☒ Life sciences ☐ Behavioural & social sciences ☐ Ecological, evolutionary & environmental sciences

For a reference copy of the document with all sections, see nature.com/documents/nr-reporting-summary-flat.pdf

Life sciences study design

All studies must disclose on these points even when the disclosure is negative.

Sample size	Minimum number of samples/animals/patients to obtain a power of 85% to assess changes of >15% between groups was calculated using ssize.fdr package in R/Bioconductor based on previous experiments. Sample size equal or greater than 3 for all analyses.
Data exclusions	Tissue cores from the commercially purchased pulmonary fibrosis tissue microarray were excluded from the analysis if the tissue was less than 75% fibrotic based on pathologist assessment. No other data was excluded.
Replication	All experiments in this study were at least repeated twice with all attempts at replication were successful.
Randomization	Randomization was performed for wild-type and Epm2a/GAA knockout mice treated with saline (control) or bleomycin. NO other randomization was performed. Human fibrosis samples were analyzed individually and presented as such and did not need randomization.
Blinding	All experiments were performed in a blinded fashion without previous knowledge of groups/identifiers.

Reporting for specific materials, systems and methods

We require information from authors about some types of materials, experimental systems and methods used in many studies. Here, indicate whether each material, system or method listed is relevant to your study. If you are not sure if a list item applies to your research, read the appropriate section before selecting a response.

Materials & experimental systems

n/a	Involved in the study	
☐	☒	Antibodies
☒	☐	Eukaryotic cell lines
☒	☐	Palaeontology and archaeology
☐	☒	Animals and other organisms
☒	☐	Clinical data
☒	☐	Dual use research of concern

Methods

n/a	Involved in the study	
☒	☐	ChIP-seq
☒	☐	Flow cytometry
☒	☐	MRI-based neuroimaging

Antibodies

Antibodies used	GAA rabbit (Proteintech 14267-1-AP), LAMP2 rabbit (Abcam ab125068), a-SMA Rabbit (GeneTex GTX100034), Goat anti-rabbit Alexa488 (thermo A-11034), Goat anti-mouse Alexa647 (Thermo A-21236), Glycogen (IV58b6) mouse (in house)
Validation	Glycogen antibody was validated against tissue sections undergo amylase treatment that ablate all glycogen within the tissue.

Validation	All other antibodies were routinely used antibodies validated by the manufacturer's for histochemical staining against human and mouse.

Animals and other research organisms

Policy information about studies involving animals; ARRIVE guidelines recommended for reporting animal research, and Sex and Gender in Research

Laboratory animals	Wild-type and Epm2a/Gaa knockout C57BL/6J (mus musculus) at 3-6 months of age were used in this study.
Wild animals	The study did not involve wild animals.
Reporting on sex	Both male and female mice were used in the study and no sex differences were observed.
Field-collected samples	The study did not involve samples collected from the field.
Ethics oversight	The University of Kentucky Institutional Animal Care and Use Committee has approved all of the animal procedures in this study.

Note that full information on the approval of the study protocol must also be provided in the manuscript.
